# Fungal pathogens causing postharvest anthracnose and fruit rot in Indian jujube (*Ziziphus mauritiana*) from northern Thailand and their fungicide response profiles

**DOI:** 10.3389/fpls.2025.1634557

**Published:** 2025-07-10

**Authors:** Wipornpan Nuangmek, Nakarin Suwannarach, Sahattaya Sukyai, Bunruam Khitka, Jaturong Kumla

**Affiliations:** ^1^ School of Agriculture and Natural Resources, University of Phayao, Phayao, Thailand; ^2^ Office of Research Administration, Chiang Mai University, Chiang Mai, Thailand; ^3^ Center of Excellence in Microbial Diversity and Sustainable Utilization, Chiang Mai University, Chiang Mai, Thailand; ^4^ Department of Biology, Faculty of Science, Chiang Mai University, Chiang Mai, Thailand

**Keywords:** crop plant, fungal disease, fungicide response, pathogen identification, postharvest disease

## Abstract

Indian jujube (*Ziziphus mauritiana*) is an economically important crop in Thailand. During 2024–2025, anthracnose and rot symptoms were observed on postharvest Indian jujube fruits in northern Thailand. Thus, this study aimed to identify the causal agents and evaluate their fungicide response. Nine fungal strains were isolated and identified as *Colletotrichum fructicola*, *C. siamense*, and *Fusarium weifangense* through morphological and multi-gene phylogenetic analyses. Pathogenicity tests confirmed *C. fructicola* and *C. siamense* cause postharvest anthracnose, while *F. weifangense* causes fruit rot. Fungicide assays revealed that *C. siamense* was completely inhibited by copper oxychloride and copper hydroxide, with captan, cyproconazole, difenoconazole + azoxystrobin, and difenoconazole also showing effectiveness against most strains. *Colletotrichum fructicola* was most effectively inhibited by difenoconazole, although no fungicide achieved complete inhibition. *Fusarium weifangense* was fully inhibited by carbendazim, copper hydroxide, and cyproconazole, but was not suppressed by azoxystrobin. This represents the first report of *C. fructicola* and *C. siamense* causing anthracnose in Indian jujube in Thailand, and the first worldwide report of *F. weifangense* causing fruit rot in this crop. These findings provide crucial insights for developing effective management strategies against postharvest diseases of Indian jujube.

## 1 Introduction

Indian jujube (*Ziziphus mauritiana* Lam.), belonging to the family Rhamnaceae, is a tropical fruit tree native to the Indian subcontinent, but it is widely cultivated across different regions of Africa, Asia, and the Middle East (Janick and Paull, 2008; Pareek and Yahia, 2013; Bebawi et al., 2016; O’Brien et al., 2023). Some common English names for this tree are ber, chinee apple, chinese date, chinese bear tree, common jujube, and desert apple. The Indian jujube fruits are globose to oval-shaped and come in various colors, including green, yellow, and red, depending on ripening stage. Typically, this fruit is not only eaten fresh but is also used in various processed forms, including dried, juiced, and pickled. Indian jujube fruits are high in antioxidants, bioactive compounds, and nutrients, making them an excellent food source (Prakash et al., 2020; Muhammad et al., 2022; Sharif et al., 2022). Additionally, the jujube tree’s bark, leaves, and roots are used in traditional medicine due to their potential therapeutic benefits, which include antibacterial, anti-inflammatory, and antioxidant properties (Naaz et al., 2020; Prakash et al., 2020; Butt et al., 2021). Jujube is cultivated in Southeast Asia (Cambodia, Laos, Myanmar, Thailand, and Vietnam), South Asia (Bangladesh, India, Nepal, and Pakistan), East Asia (northern and western China and Taiwan), and Central Asia (Afghanistan, Iran, Iraq, Oman, and the United Arab Emirates) (Pareek and Yahia, 2013; Bebawi et al., 2016; O’Brien et al., 2023). Indian jujube is susceptible to several diseases that significantly affect its production, quality, and postharvest longevity. Among the most common diseases are anthracnose of fruit, caused by *Colletotrichum* species and fruit rot caused by various fungal pathogens, such as *Alternaria*, *Aspergillus*, *Cladosporium*, *Diaporthe*, *Fusarium*, *Neofusicoccum*, *Penicillium*, and *Phomopsis* species (Athipunyakom et al., 2010; Mirzaee, 2014; Misra et al., 2013; Alam et al., 2017; Han et al., 2023a; Won et al., 2023; Xu et al., 2023). These pathogens can infect fruits during different developmental stages, often causing premature fruit drop and postharvest storage damage, which ultimately compromises the fruits’ marketability, shortens their shelf life, and significantly reduces their economic value. Fungicide application remains a cornerstone of integrated disease management for fungal pathogens in fruit crops, with several fungicide classes demonstrating variable modes of action and efficacy against fungal pathogens (El-Baky and Amara, 2021; FRAC, 2024). Several fungicide groups, e.g., demethylation inhibitors (cyproconazole, propiconazole, and triticonazole), phenylamides (metalaxyl), succinate dehydrogenase inhibitors (boscalid, carboxin, and fludioxonil), quinone outside inhibitors (azoxystrobin and famoxadone), benzimidazole (carbendazim), and dithiocarbamates (mancozeb and maneb) have been used for control and management of plant diseases caused by fungal species with different modes of action (Zubrod et al., 2019; Bachkar et al., 2021; Yin et al., 2023; FRAC, 2024). Moreover, copper-based fungicides (copper sulfate, copper hydroxide, and copper oxychloride) have been widely used to control plant fungal diseases due to their broad-spectrum activity, and they have demonstrated strong inhibitory effects against multiple strains of fungal pathogens, including species of *Alternaria*, *Botrytis*, *Colletotrichum*, and *Fusarium* (Pariona et al., 2019; Yuan et al., 2023; Grzanka et al., 2024). Therefore, precise disease diagnosis is essential, requiring the development of comprehensive integrated disease management strategies that encompass resistant cultivar selection, optimized fungicide application techniques, and exploration of alternative control methods.

In Thailand, the cultivation of Indian jujube has gained significant economic value due to the increasing demand for this fruit. It is primarily cultivated in the central region (Kanchanaburi, Samut Sakhon, and Suphan Buri Provinces) and in the northeastern region (Kalasin Province). Additionally, areas in northern Thailand where Indian jujube is cultivated include Chiang Rai, Chiang Mai, Phayao, and Sukhothai Provinces. Currently, the area under Indian jujube cultivation is expanding, as it has become one of the most important crops for the economy. However, planting Indian jujube in unsuitable areas, along with climate change, can make both trees and fruit more susceptible to diseases. Prior to this study, *Alternaria alternata*, *Pseudocercospora jujubae*, *Guignardia* sp., *Mycosphaerella* sp., *Pestalotiopsis* sp., and *Phyllosticta* sp. are the causative agents of leaf spot disease, while *Colletotrichum gloeosporioides* was associated with anthracnose and fruit rot diseases in Indian jujube in Thailand (Athipunyakom et al., 2010). However, research on the accurate diagnosis of anthracnose and fruit rot diseases in Indian jujube in Thailand has been limited. Thus, further investigations remain crucial to identify additional pathogens associated with these diseases within the country. In this study, sympotms of anthracnose and fruits rot on Indian jujube fruits were observed throughout the postharvest period from November 2024 to January 2025 in Chiang Mai and Phayao Provinces, northern Thailand. The purpose of this study is to isolate and identify the causal agents associated with observed symptoms using morphological characteristics and molecular phylogenetic analyses. The pathogenicity of the obtained fungal isolates will be evaluated by applying Koch’s postulates. Furthermore, the growth response of the isolated fungal strains to various commercial fungicides was determined.

## 2 Materials and methods

### 2.1 Sample collection

During the postharvest storage period from November 2024 to January 2025, Indian jujube fruits (*Ziziphus mauritiana*) exhibiting characteristic anthracnose and fruit rot symptoms were collected from local markets across Chiang Mai and Phayao Provinces in northern Thailand. Ten fruits of each location were collected and transported to the laboratory within 24 h. Symptomatic fruits were examined using a Nikon H550S stereo microscope (Tokyo, Japan) and subsequently placed in a plastic box lined with moistened filter paper to promote conidial production. Conidia were examined under a Nikon Eclipse Ni-U light microscope (Tokyo, Japan) and subsequently used for further fungal isolation.

### 2.2 Fungal isolation and morphological studies

The causal fungi were isolated from diseased lesions using the single-conidial isolation method (Choi et al., 1999) on 1.0% water agar supplemented with 0.5 mg/L streptomycin. Following a 24- to 48-hour incubation at 25°C in darkness, individual germinated conidia were transferred onto potato dextrose agar (PDA; CONDA, Madrid, Spain) supplemented with 0.5 mg/L streptomycin, utilizing a stereo microscope. Each pure fungal culture was deposited in the Sustainable Development of Biological Resources (SDBR) Laboratory’s Culture Collection within the Faculty of Science at Chiang Mai University in Thailand. The colony characteristics were examined on PDA after incubation at 25°C in the dark for one week. Microscopic structures were examined using a light microscope. The Tarosoft (R) Image Frame Work program was used to measure the size of fungal structures (e.g., chlamydospore, conidiophore, conidiogenous cell, and conidia).

### 2.3 DNA extraction, amplification, and sequencing

Fungal strains were cultured on PDA for five days at 25°C under dark conditions. Subsequently, the fungal mycelia were collected and used for DNA extraction, following the protocol outlined by Suwannarach et al. (2024). The DNA Extraction Mini Kit (FAVORGEN, Taiwan) was used to extract DNA following the manufacturer’s procedures. The amplification of the internal transcribed spacer (ITS), actin (*ACT*), beta-tubulin (*TUB*), calmodulin (*CAL*), glyceraldehyde-3-phosphate dehydrogenase (*GAPDH*), RNA polymerase II second largest subunit (*RPB2*), and translation elongation factor 1-alpha (*TEF1*-*α*) genes carried out using the primer pairs ITS4/ITS5, ACT512F/ACT738R, T1/T2, CL1C/CL2C or CAL-228F/CAL-2Rd, GDF1/GPDHR2, RPB2-5F2/RPB2-7cR, and EF1/EF2, following the previous studies (Cai et al., 2009; Wang et al., 2019; Crous et al., 2021; Bhunjun et al., 2021; Norphanphoun and Hyde, 2023). The PCR products were individually purified using the NucleoSpin^®^ Gel and PCR Clean-up Kit (Macherey-Nagel, Germany) following the manufacturer’s instructions. Sequencing reactions were performed by 1st Base Company Limited, Selangor, Malaysia.

### 2.4 Sequence alignment and phylogenetic analyses

Sequence similarity searches were conducted using the BLAST program, available through NCBI (http://blast.ddbj.nig.ac.jp/top-e.html, accessed on 5 April 2025). Comprehensive sequence datasets were compiled, incorporating newly generated sequences along with reference sequences obtained from GenBank database and previous studies. The MUSCLE software (Edgar, 2004) was employed for the alignment of multiple sequences, and BioEdit version 6.0.7 (Hall, 2004) was used for any necessary adjustments. Maximum likelihood (ML) analysis was carried out for phylogenetic analyses on the GTRCAT model with 1000 bootstrap (BS) replications using RAxML-HPC2 version 8.2.12 via the CIPRES web platform (Felsenstein, 1985; Stamatakis, 2006). The resulting phylogenetic trees were visualized using FigTree v1.4.0 (Rambaut, 2019).

### 2.5 Pathogenicity tests

Healthy commercial Indian jujube fruits were surface sterilized by soaking in a 1.0% (*v*/*v*) NaClO solution for 5 min, rinsed three times with sterile distilled water, and subsequently air-dried for 10 min at room temperature (25 ± 2°C). Afterward, a uniform wound (5 pores, each 1 mm deep and 0.5 mm wide) was made at the equator of each fruit using aseptic needles. Nine fungal strains (SDBR-CMU527 to SDBR-CMU535) were obtained and used in this experiment. Conidia from each fungal strain were collected from two-week-old PDA cultures and suspended in sterile distilled water, adjusted to a concentration of 1 × 10^6^ conidia/mL using a hemocytometer. A 10 µL conidial suspension of each fungal strain was applied to the wounded areas of the fruits. For the control treatment, the wounded fruits were treated with sterile distilled water instead of the conidial suspension. After inoculation, fruits from each treatment were placed in individual sterile 4 L plastic boxes with a relative humidity of 78 ± 2%. The boxes were maintained at 25°C. Each treatment was carried out with 10 replications, performed independently twice under the same conditions. Lesion diameters caused by each fungal strain were measured at five days after inoculation, and disease progression was monitored for 15 days. Using the single conidial isolation method, the fungi were re-isolated from the infected fruits and subsequently identified, thereby fulfilling Koch’s postulates.

### 2.6 Effect of commercial fungicides against fungal pathogens

Ten different fungicides, all of which are commercially available and approved for agricultural use in Thailand, were selected for investigation in this study. Each fungicide was applied according to the manufacturer’s recommended concentrations and application guidelines. The information, trade names, group names, and recommended concentrations of each fungicide are presented in **Table 1**. Each fungicide was prepared and added to an autoclaved PDA to obtain the final concentration according to recommended concentrations. Each fungal strain was inoculated into the tested agar media using a mycelial plug (5 mm in diameter) from a colony that had been cultured on PDA for five days. PDA without any fungicide was used as the control treatment. The plates were incubated in darkness at 25°C for one week, or until the control plates were fully covered with fungal mycelia. After that, the colony diameter of each fungal strain in each fungicide treatment was measured and compared to the control treatment. The percentage of growth inhibition (PGI) was calculated using the formula provided by Pandey et al. (2024): PGI = (C − T)/C × 100, where C and T represent the colony diameter (in mm) in control and fungicide treatment, respectively. Subsequently, PGI value was categorized as completely inhibited (100% inhibition), effective (≥50% inhibition), or ineffective (<50% inhibition). Three replicates were performed for each fungal strain and fungicide, with the experiment repeated independently twice under identical conditions.

**Table 1:** Information, trade names, group names, and recommended concentrations of fungicides used in this study.

Fungicide name	Trade name	Group name	Recommended concentration (ppm)
Azoxystrobin	Amistar^®^	Quinone outside inhibitor	125
Benalaxyl-M (4%) + mancozeb (65%)	Fantic M WG^®^	Phenylamide + Dithiocarbamate	80 + 1300
Captan	Captan 50^®^	Phthalimides	1000
Carbendazim	Dazine^®^	Benzimidazole	750
Copper hydroxide	Funguran-OH^®^	Inorganic copper-based	1540
Copper oxychloride	Copina 85 WP^®^	Inorganic copper-based	1700
Cyproconazole	Alto^®^	Demethylation inhibitors	60
Difenoconazole	Score^®^	Demethylation inhibitors	187
Difenoconazole (12.5%) + azoxystrobin (20%)	Ortiva^®^	Demethylation inhibitor **+** Quinone outside inhibitor	240 + 385
Mancozeb	Newthane M-80^®^	Dithiocarbamate	1300

### 2.7 Statistical analysis

The normality of data from two repeated experiments on lesion diameter and fungicide response was evaluated using the Shapiro-Wilk test in SPSS version 26, with a significance level of *p*<0.05. The results indicated non-significant findings, so the data from these repeated experiments were assessed for the assumptions of one-way analysis of variance (ANOVA). Significant differences at *p*<0.05 were then determined using Duncan’s Multiple Range Test (DMRT).

## 3 Results

### 3.1 Disease symptoms and fungal isolation

**Figure 1:**
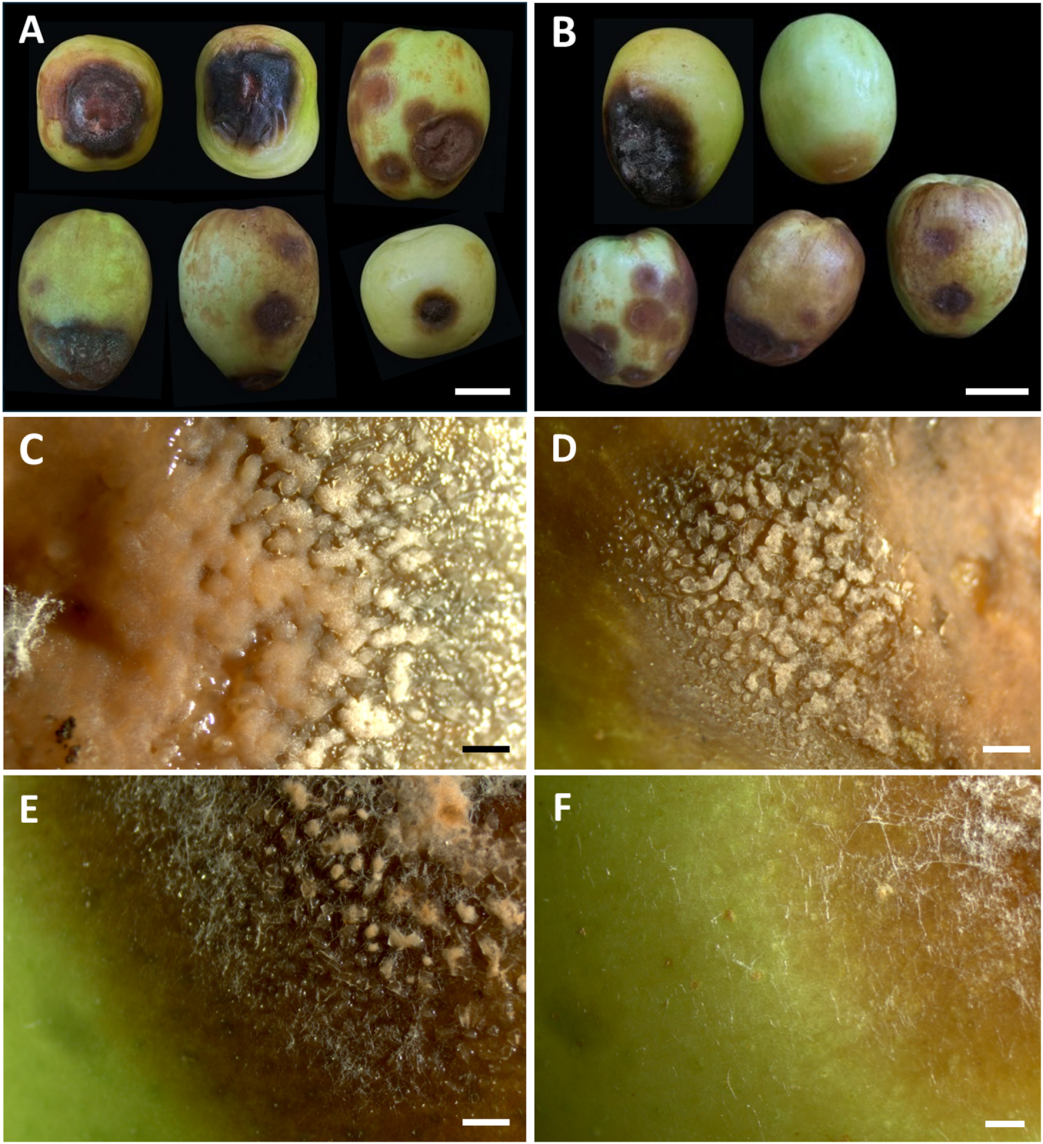
Symptoms of anthracnose and rot diseases on Indian jujube fruits during the postharvest storage period **(A, B)**. The orange conidial masses appear on the surface of the lesions **(C–E)**. White mycelium observed on decaying rot lesions **(F)**. Scale bars **(A, B)** = 20 mm, **(C–F)** = 1 mm.

In Chiang Mai Province, disease incidence ranged from 15% to 20% per 100 fruits during postharvest storage, with temperatures between 25 to 35°C and relative humidity of 60% to 75% over a 7 to 14-day period. Conversely, in Phayao Province, the disease incidence was slightly higher, spanning 20 to 25% per 100 fruits, under storage conditions of 27 to 35°C and 60% to 70% relative humidity over a 7 to 14-day period. On individual fruits, disease severity was characterized by lesions covering 20% to 60% of the total surface area. Lesions or spots initially appearing on the fruits range in color from light to dark brown. The lesions are typically irregular in shape and enlarge over time. The affected areas turn dark brown or black as the disease progresses. As the disease progresses, the fruit tissues beneath the lesions become soft, water-soaked, and mushy. Finally, the fruit decays and rots completely (**Figures 1A, B**). For anthracnose disease, a light orange to orange conidial mass appears on the surface of the lesions under moist conditions (**Figures 1C–E**). Meanwhile, white mycelium with conidiation was found on some decaying fruits (**Figure 1F**). Nine fungal strains were obtained, and pure cultures of each strain were deposited in the SDBR Laboratory Culture Collection under number SDBR-CMU527 to SDBR-CMU535. Eight strains were obtained from anthracnose disease samples, including five strains (SDBR-CMU527 to SDBR-CMU531) from Chiang Mai Province and three strains (SDBR-CMU532 to SDBR-CMU534) from Phayao Province. The remaining strain (SDBR-CMU535) was obtained from a fruit rot disease sample collected from Phayao Province.

### 3.2 Fungal identification

The eight fungal strains isolated from anthracnose symptoms were initially identified as morphologically related to the genus *Colletotrichum* and were divided into two groups based on morphological similarities. The first group contained three strains (SDBR-CMU527, SDBR-CMU531, and SDBR-CMU532), while the second group contained five strains (SDBR-CMU528, SDBR-CMU529, SDBR-CMU530, SDBR-CMU533, and SDBR-CMU534). Additionally, the strain SDBR-CMU535, which was isolated from a fruit rot symptom, was identified based on morphological characteristics as belonging to the genus *Fusarium*. Subsequently, these nine fungal strains were identified through multi-gene molecular phylogenetic analyses.

**Figure 2:**
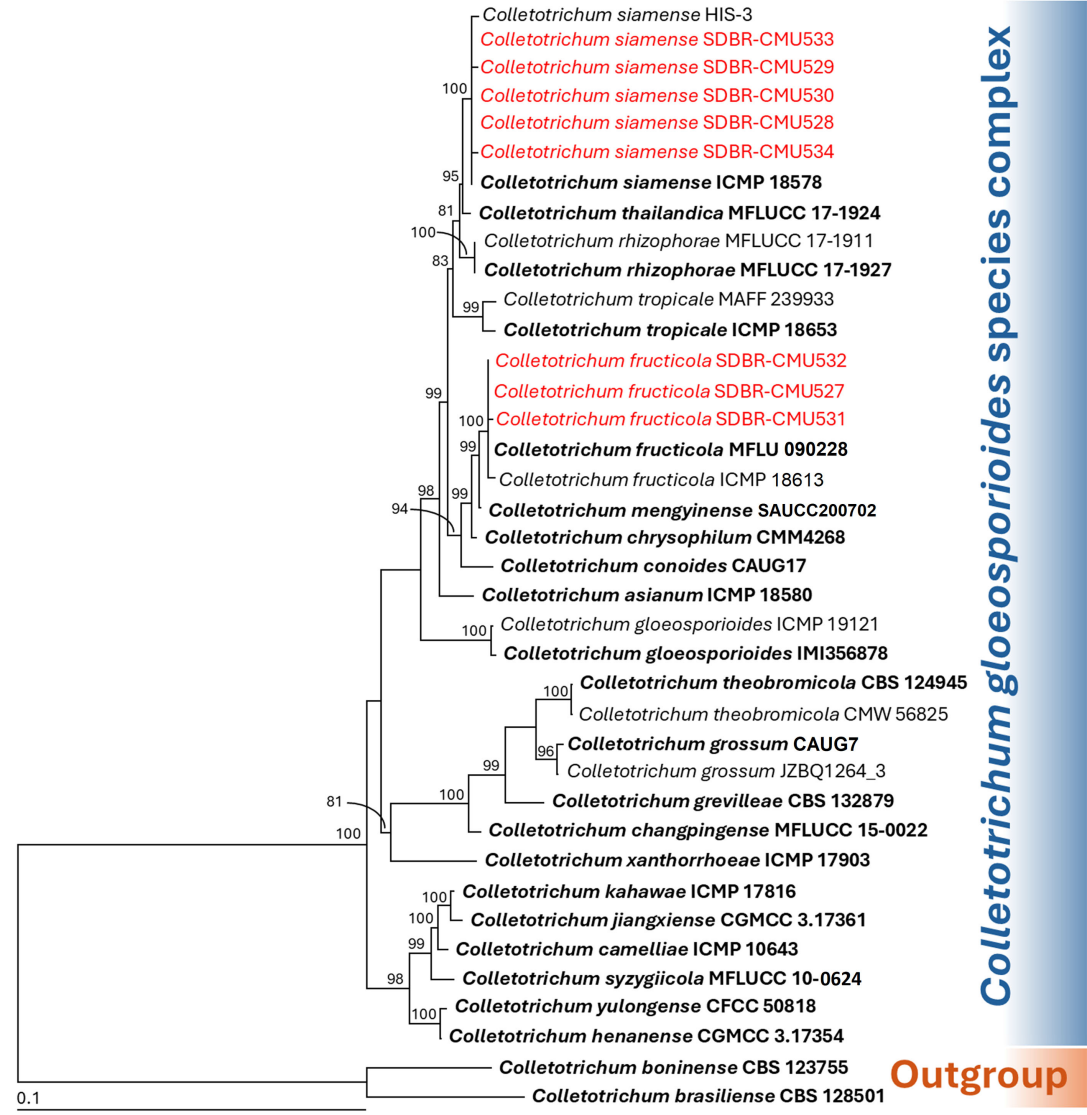
Maximum likelihood phylogenetic tree of 38 fungal strains of the combined ITS, *GAPDH*, *CAL*, *ACT*, and *TUB* sequences. *Colletotrichum boninense* CBS 128501 and *C. brasiliense* CBS 123755 were designated as the outgroup. Bootstrap support (BS) ≥ 70% are reported on the branches. The scale bar represents the expected number of nucleotide substitutions per site. Type strains are shown in bold, and the fungal strains obtained in this study are indicated in red.

Phylogenetic identification of *Colletotrichum* species employed a five-gene analysis approach using ITS, *GAPDH*, *CAL*, *ACT*, and *TUB* genes, consistent with established methodologies from previous studies (Bhunjun et al., 2021; Mu et al., 2021; Norphanphoun and Hyde, 2023). The sequences obtained from each fungal strain were deposited in the GenBank database, and the results from BLAST are presented in **Table 2**. The complete aligned dataset was 2441 bp including gaps and contained sequences from 38 different taxa. This dataset combined five different genetic regions: ITS (positions 1–581), *GAPDH* (positions 582–875), *CAL* (positions 876–1609), *TUB* (positions 1609–2290), and *ACT* (positions 2291–2441). *Colletotrichum boninense* species complex (*C*. *boninense* and *C. brasiliense*) was set as outgroup. There are 725 distinct alignment patterns in the sequence, and 14.37% of undetermined characters or gaps. A final ML optimization likelihood value of -8580.1872 was obtained. The phylogenetic tree successfully placed eight *Colletotrichum* strains within the *C. gloeosporioides* species complex (**Figure 2**). Three strains from the first group clustered within the *C. fructicola* clade, alongside the type strain MFLU 090228. This clade exhibited 100% bootstrap support and showed a close phylogenetic relationship to *C. mengyinense*. Additionally, five strains in the second group belonged to the *C. siamense* clade, which included the type strain ICMP 18578 with a 99% BS value. *Colletotrichum siamense* formed a sister taxon to *C. thailandicum.*

**Table 2:** GenBank accession numbers and BLAST results revealed the highest sequence similarity between the fungal strains in this study and the holotype species available in the GenBank database.

Fungal strain	Gene	GenBank accession number	The closely related holotype fungal species/similarity value (%)
SDBR-CMU527	ITS	PV639623	*Colletotrichum fructicola* MFLU 090228/100.00
	*GAPDH*	PV646654	*Colletotrichum fructicola* MFLU 090228/99.28
	*CAL*	PV646662	*Colletotrichum fructicola* MFLU 090228/100.00
	*ACT*	PV646670	*Colletotrichum fructicola* MFLU 090228/99.61
	*TUB*	PV646678	*Colletotrichum fructicola* MFLU 090228/100.00
SDBR-CMU528	ITS	PV639624	*Colletotrichum thailandica* MFLUCC 17-1924/100.00
	*GAPDH*	PV646655	*Colletotrichum siamense* ICMP 18578/98.48
	*CAL*	PV646663	*Colletotrichum siamense* ICMP 18578/99.73
	*ACT*	PV646671	*Colletotrichum siamense* ICMP 18578/99.61
	*TUB*	PV646679	*Colletotrichum rhizophorae* MFLUCC 17-1927/99.59
SDBR-CMU529	ITS	PV639625	*Colletotrichum siamense* ICMP 18578/98.80
	*GAPDH*	PV646656	*Colletotrichum siamense* ICMP 18578/97.99
	*CAL*	PV646664	*Colletotrichum rhizophorae* MFLUCC 17-1927/99.60
	*ACT*	PV646672	*Colletotrichum siamense* ICMP 18578/99.61
	*TUB*	PV646680	*Colletotrichum siamense* ICMP 18578/99.54
SDBR-CMU530	ITS	PV639626	*Colletotrichum siamense* ICMP 18578/98.82
	*GAPDH*	PV646657	*Colletotrichum siamense* ICMP 18578/99.28
	*CAL*	PV646665	*Colletotrichum siamense* ICMP 18578/99.87
	*ACT*	PV646673	*Colletotrichum siamense* ICMP 18578/99.43
	*TUB*	PV646681	*Colletotrichum rhizophorae* MFLUCC 17-1927/99.43
SDBR-CMU531	ITS	PV639627	*Colletotrichum fructicola* MFLU 090228/100.00
	*GAPDH*	PV646658	*Colletotrichum fructicola* MFLU 090228/99.26
	*CAL*	PV646666	*Colletotrichum fructicola* MFLU 090228/100.00
	*ACT*	PV646674	*Colletotrichum fructicola* MFLU 090228/99.61
	*TUB*	PV646682	*Colletotrichum fructicola* MFLU 090228/100.00
SDBR-CMU532	ITS	PV639628	*Colletotrichum fructicola* MFLU 090228/100.00
	*GAPDH*	PV646659	*Colletotrichum fructicola* MFLU 090228/99.28
	*CAL*	PV646667	*Colletotrichum fructicola* MFLU 090228/100.00
	*ACT*	PV646675	*Colletotrichum fructicola* MFLU 090228/99.61
	*TUB*	PV646683	*Colletotrichum fructicola* MFLU 090228/100.00
SDBR-CMU533	ITS	PV639629	*Colletotrichum siamense* ICMP 18578/98.82
	*GAPDH*	PV646660	*Colletotrichum siamense* ICMP 18578/99.28
	*CAL*	PV646668	*Colletotrichum siamense* ICMP 18578/99.87
	*ACT*	PV646676	*Colletotrichum siamense* ICMP 18578/99.43
	*TUB*	PV646684	*Colletotrichum rhizophorae* MFLUCC 17-1927/99.43
SDBR-CMU534	ITS	PV639630	*Colletotrichum siamense* ICMP 18578/98.80
	*GAPDH*	PV646661	*Colletotrichum siamense* ICMP 18578/97.99
	*CAL*	PV646669	*Colletotrichum rhizophorae* MFLUCC 17-1927/99.60
	*ACT*	PV646677	*Colletotrichum siamense* ICMP 18578/99.61
	*TUB*	PV646685	*Colletotrichum siamense* ICMP 18578/99.54
SDBR-CMU535	*CAL*	PV646686	*Fusarium weifangense* LC18333/99.82
	*RPB2*	PV646687	*Fusarium weifangense* LC18333/99.71
	*TEF1-α*	PV646688	*Fusarium weifangense* LC18333/100.00

**Figure 3:**
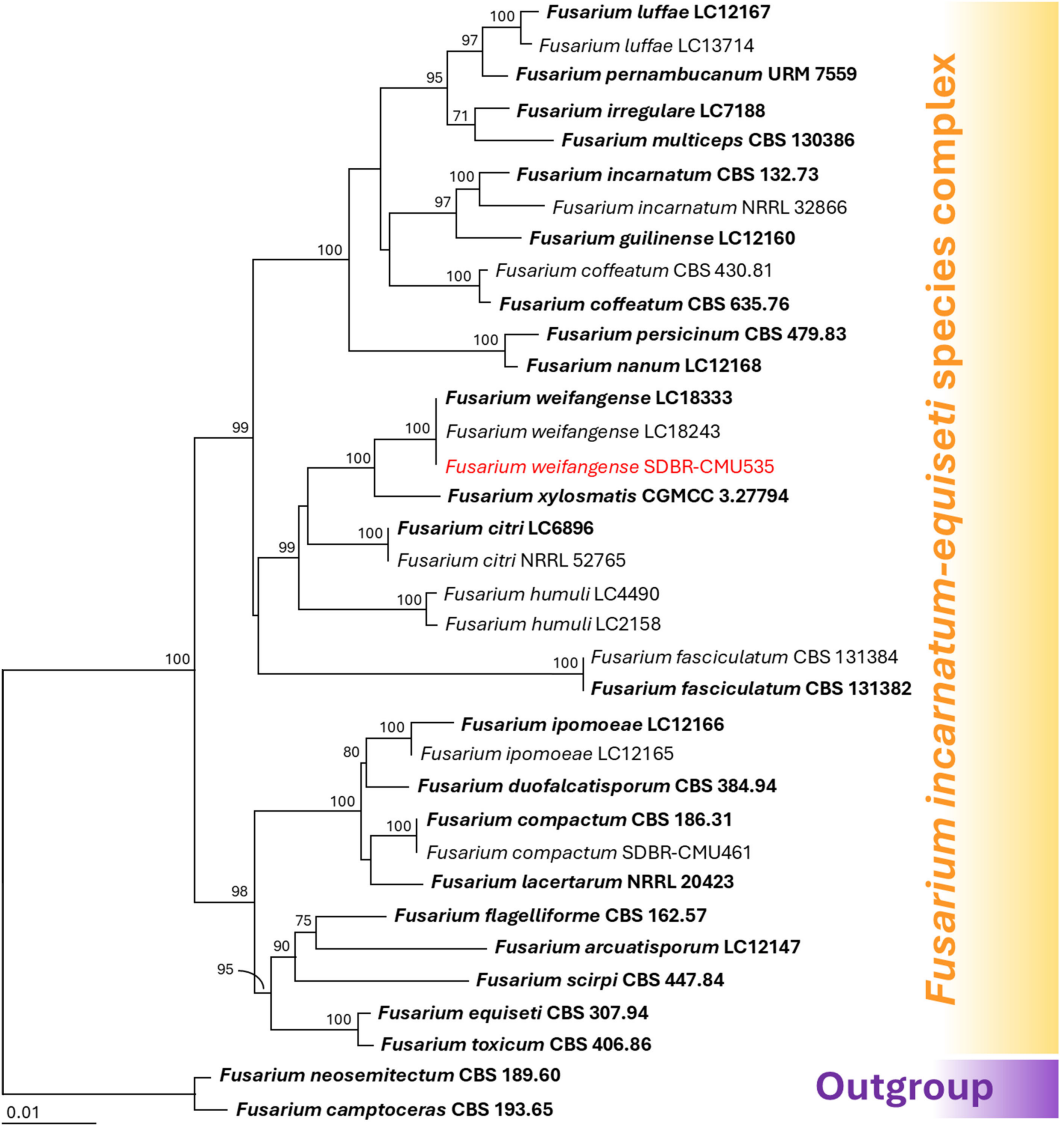
Maximum likelihood phylogenetic tree of 35 fungal strains of the combined *CAL*, *RPB2*, and *TEF1-α* sequences. *Fusarium camptoceras* and *F. neosemitectum* were designated as the outgroup. Bootstrap support (BS) ≥ 70% are reported on the branches. The scale bar represents the expected number of nucleotide substitutions per site. Type strains are shown in bold, and the fungal strains obtained in this study are indicated in red.

Species identification of *Fusarium* was conducted through multi-gene phylogenetic analyses utilizing the *CAL*, *RPB2*, and *TEF1*-*α* genes, following methodologies established in previous studies (Wang et al., 2019; Crous et al., 2021; Nuangmek et al., 2023; Suwannarach et al., 2024). The complete aligned dataset was 1844 bp including gaps and contained sequences from 35 different taxa. This dataset combined three different genetic regions: *CAL* (positions 1–533), *RPB2* (positions 534–1258), and *TEF1*-*α* (positions 1259–1844). *Fusarium camptoceras* species complex (*F. camptoceras* and *F. neosemitectum*) was used as the outgroup. There are 372 distinct alignment patterns in the sequence, and 2.14% of undetermined characters or gaps. A final ML optimization likelihood value of -6307.3702 was obtained. A phylogenetic tree is presented in **Figure 3**. *Fusarium* sp. strain SDBR-CMU535 obtained in this study was placed within *F. incanatum-equiseti* species complex. It was positioned within the *F. weifangense* clade, clustering with the type strain LC18333 and supported by a 100% bootstrap value. *Fusarium weifangense* formed a sister taxon to *F. xylosmatis*.

Through comprehensive multi-gene molecular phylogenetic analyses and detailed morphological characteristics (described below), we identified the species collected in this study as *C. fructicola* (SDBR-CMU527, SDBR-CMU531, and SDBR-CMU532), *C. siamense* (SDBR-CMU528, SDBR-CMU529, SDBR-CMU530, SDBR-CMU533, and SDBR-CMU534), and *F. weifangense* (SDBR-CMU535).

### 3.3 Morphological description of fungal pathogens

#### 3.3.1 Colletotrichum fructicola

**Figure 4:**
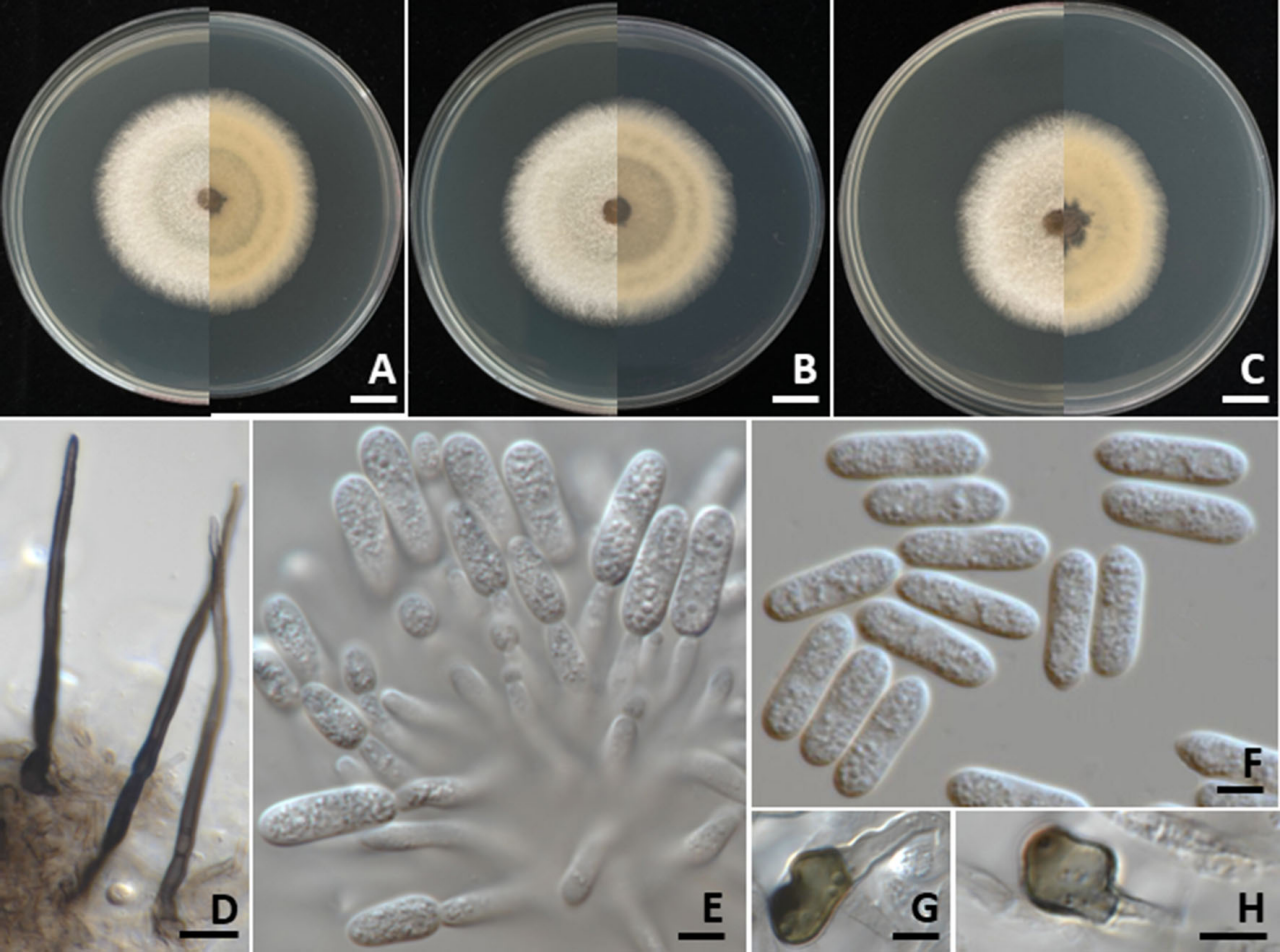
*Colletotrichum fructicola*. Colony morphology of strains SDBR-CMU527 **(A)**, SDBR-CMU531 **(B)**, and SDBR-CMU532 **(C)** grown on potato dextrose agar after one week of incubation at 25 °C (left, surface view and right, reverse view). Micromorphology of strains SDBR-CMU527 **(D–H)**. Setae **(D)**. Conidiogenous cells giving rise to conidia **(E)**. Conidia **(F)**. Appressoria **(G, H)**. Scale bars: **(A–C)** = 10 mm; **(D)** = 10 µm, **(E–H)** = 5 µm.

Colonies on PDA measured 55–70 mm in diameter after one week of incubation at 25°C in the dark (**Figure 4**). Colonies were velvety, circular, with undulate margins. The surface was pale grey at the center and white at the margin, turning grey with age; the reverse side ranged from white to pale yellow. Light orange to orange conidial masses were observed. Setae were brown to dark brown, smooth-walled, 2–4-septate, and measured 35–80 µm long (*n* = 50), with tips that were either acute or obtuse. Appressoria appeared brown to dark brown, exhibited irregular shape, and measured 7–9 × 4–5 µm (*n* = 50). Conidiophores were hyaline, septate, branched, and ranged from cylindrical to inflated shape. Conidiogenous cells were clavate to cylindrical, hyaline, and measured 15–25 × 3–5 µm (*n* = 50). Conidia were smooth-walled, hyaline, aseptate, cylindrical with a rounded apex, guttulate, and measured 12–19 × 4–5 µm (*n* = 50). The morphological characteristics of the *C. fructicola* strains (SDBR-CMU527, SDBR-CMU531, and SDBR-CMU532) obtained in this study closely resembled those described for *C. fructicola* in previous studies (Prihastuti et al., 2009; Norphanphoun and Hyde, 2023).

#### 3.3.2 Colletotrichum siamense

**Figure 5:**
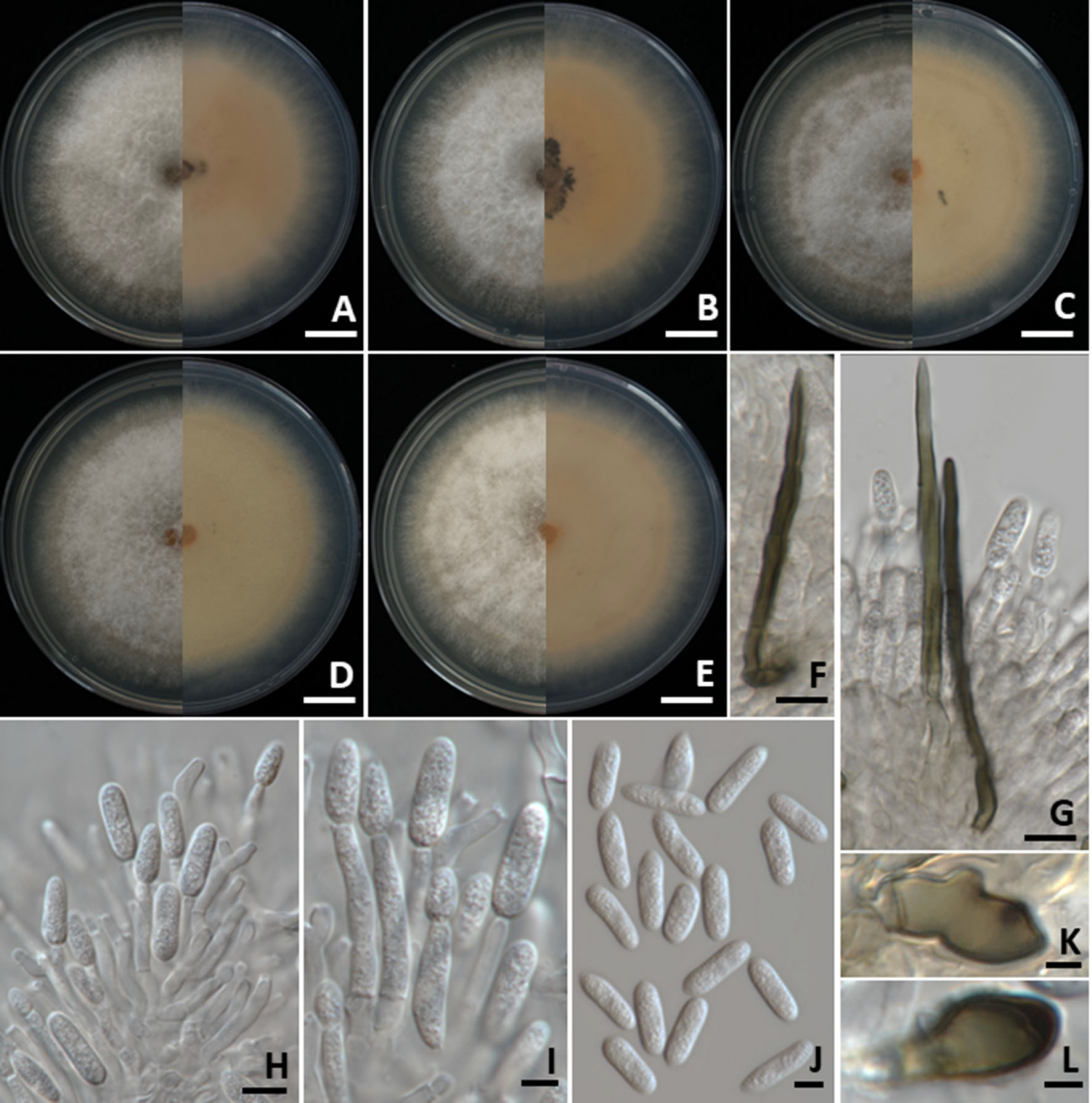
*Colletotrichum siamense*. Colony morphology of strains SDBR-CMU528 **(A)**, SDBR-CMU529 **(B)**, SDBR-CMU530 **(C)**, SDBR-CMU533 **(D)**, and SDBR-CMU534 **(E)** grown on potato dextrose agar after one week of incubation at 25 °C (left, surface view and right, reverse view). Micromorphology of strains SDBR-CMU530 **(F–L)**. Setae **(F, G)**. Conidiogenous cells giving rise to conidia **(H, I)**. Conidia **(J)**. Appressoria **(K, L)**. Scale bars: **(A–E)** = 10 mm; **(F, G)** = 10 µm, **(H–J)** = 5 µm, **(K, L)** = 1 µm.

Colonies on PDA measured 70–85 mm in diameter after one week of incubation at 25°C in the dark (**Figure 5**). Colonies were cottony and circular with undulate margins. The surface was pale grey at the center and white at the margin, becoming grey with age. The reverse side ranged from pale orange to pale brown. Light orange to orange conidial masses were observed. Setae were brown to dark brown, smooth-walled, 2–4-septate, and measured 80–120 µm long (*n* = 50), with tips that were either acute or obtuse. Appressoria exhibited brown to dark brown, showed oval or irregular shape, and ranged in size from 5–8 × 4–6 µm (*n* = 50). Conidiophores were hyaline, septate, branched, and cylindrical to clavate in shape. Conidiogenous cells were cylindrical to clavate, hyaline, and measured 15–20 × 3–5 µm (*n* = 50). Conidia were smooth-walled, hyaline, aseptate, subcylindrical to oblong in shape, rounded at the apex, guttulate, and measured 10–19 × 4–5 µm (*n* = 50). The strains of *C. siamense* obtained in this study (SDBR-CMU528, SDBR-CMU529, SDBR-CMU530, SDBR-CMU533, and SDBR-CMU534) displayed morphological characteristics that align with previously published descriptions of this species (Prihastuti et al., 2009; Li et al., 2022; Kaewchai et al., 2024).

#### 3.3.3 Fusarium weifangense

**Figure 6:**
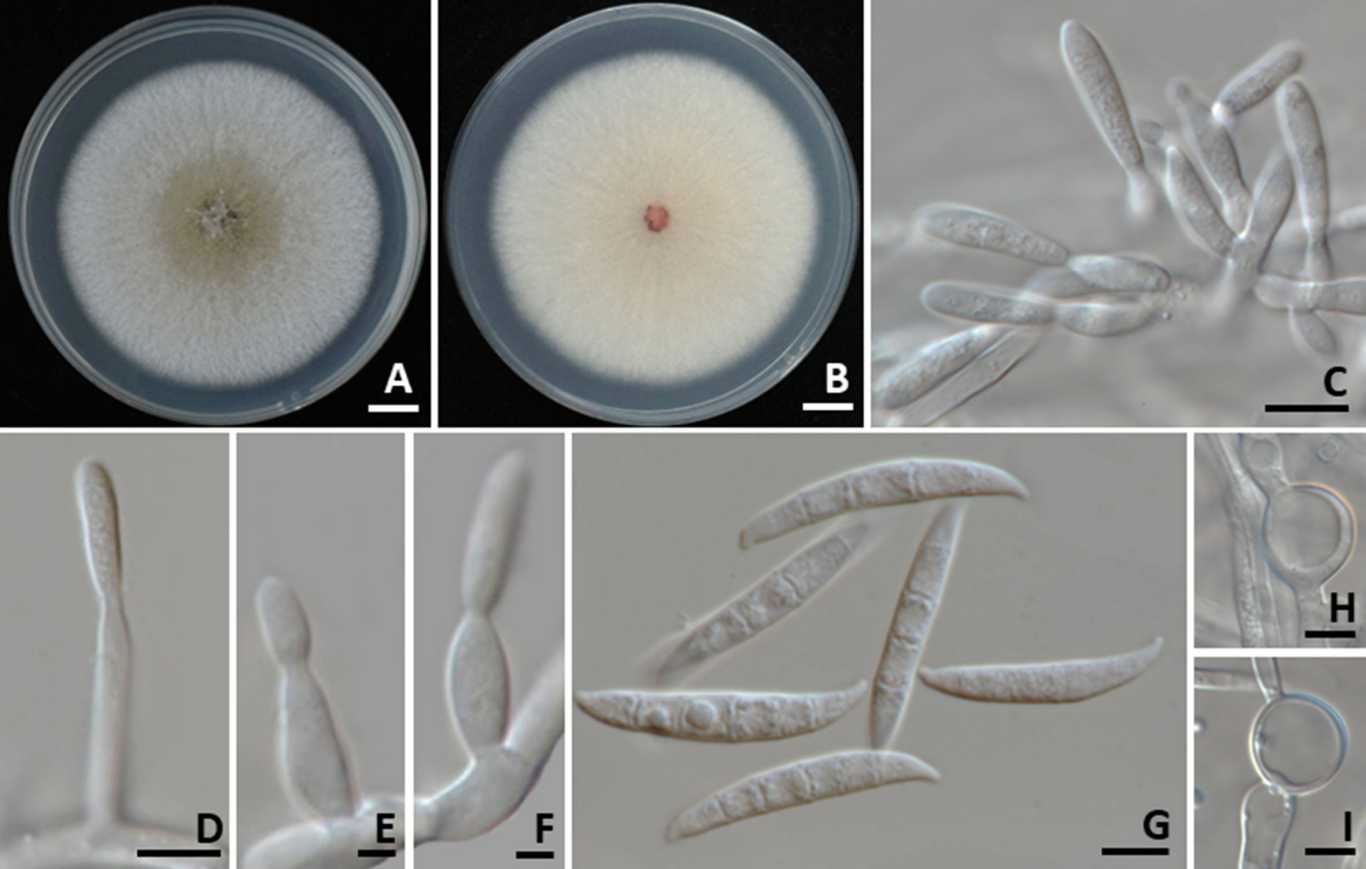
*Fusarium weifangense*. Colony morphology of strains SDBR-CMU535 grown on potato dextrose agar after one week of incubation at 25 °C. Surface view **(A)**. Reverse view **(B)**. Sporodochial conidiophores **(C)**. Aerial conidiophores **(D–F)**. Conidia **(G)**. Chlamydospores **(H, I)**. Scale bars: **(A, B)** = 10 mm; **(C, D, G–I)** = 5 µm, **(E, F)** = 1 µm.

Colonies grown on PDA in the dark reached 80–90 mm in diameter after one week of incubation at 25°C (**Figure 6**). Colonies were flat, felty to velvety with an entire margin. The surface was white, becoming yellowish-white at the center, while the reverse side was white. Sporodochia were observed. Sporodochial conidiophores were densely arranged, each bearing apical whorls of a single phialide. Sporodochial phialides are characterized as monophialidic, exhibiting a subulate to subcylindrical shape, smooth, and measured 16–20 × 2–3 μm. Conidiophores, which arise from aerial mycelium and are simplified to monophialides, exhibit a subulate to subcylindrical shape, characterized by smooth, thin-walled, hyaline, and measured 9.5–12 × 2–3 µm (*n* = 50). Chlamydospores were globose to ellipsoidal, hyaline, solitary, terminal or intercalary, 11–25 × 7.5–22 μm. Conidia were hyaline, slightly curved, tapering at both ends, with a blunt apical cell and a blunt to slightly notched basal cell, thin-walled and smooth, 1–5-septate, and measured 15–40.5 × 3–6 µm (*n* = 50). The morphological characteristics of *F. weifangense* SDBR-CMU535 identified in this study aligned closely with the descriptions provided for *F. weifangense* in the publications by Han et al. (2023b) and Ai et al. (2025). Nonetheless, this study provides the first observation of chlamydospores in *F. weifangense.*

### 3.4 Pathogenicity test

**Figure 7:**
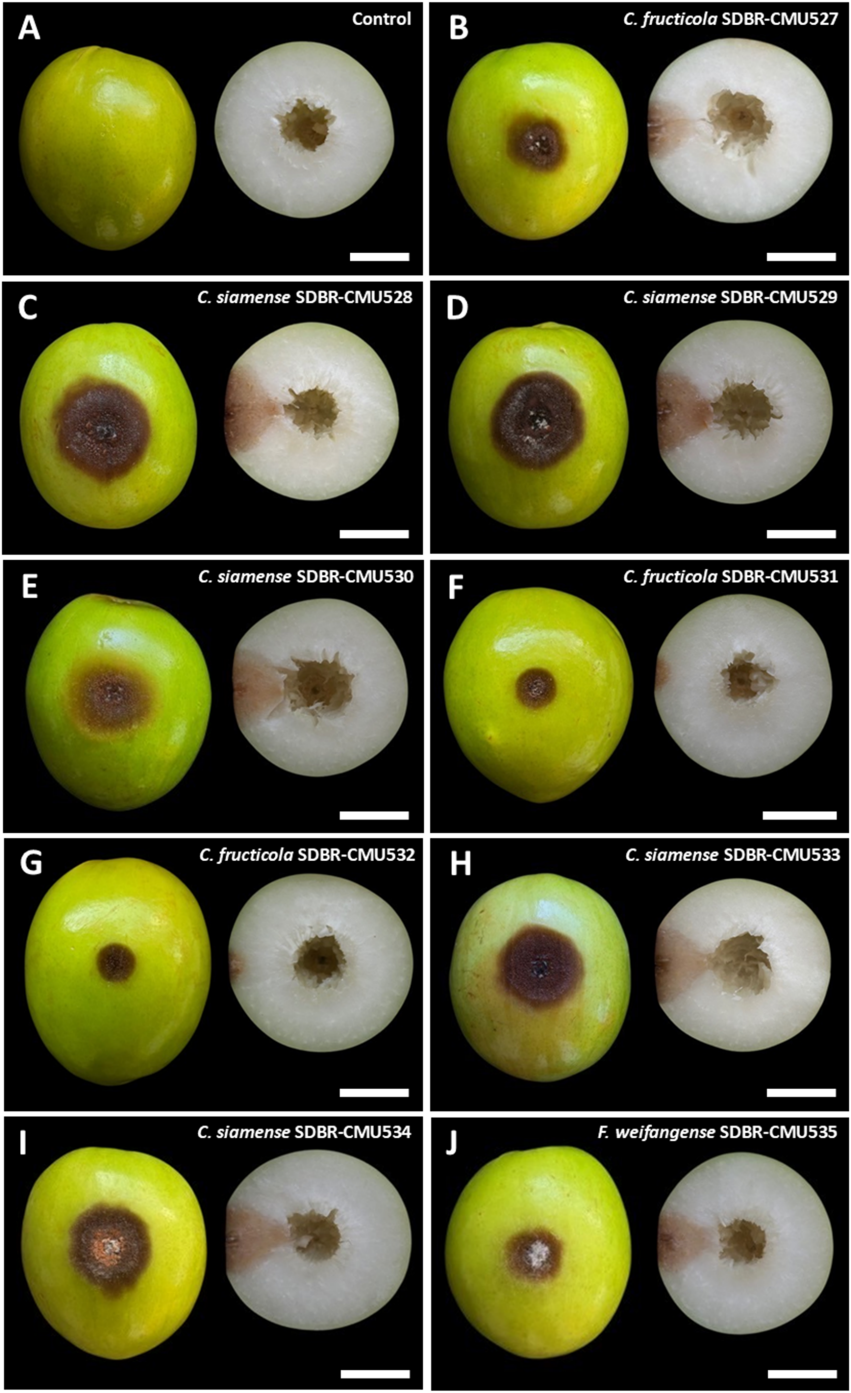
Pathogenicity test of fungal causal agents of postharvest anthracnose and fruit rot diseases on Indian jujube after five days of inoculation (left, surface view and right, cross section). Control **(A)**. Anthracnose symptoms caused by inoculation with *C. fructicola* **(B, F, G)**. Anthracnose symptoms caused by inoculation with *C. siamense* **(C, D, E, H, I)**. Fruit rot symptoms caused by inoculation with *Fusarium weifangense* **(J)**. Scale bars = 20 mm.

All fungal strains were found to be pathogenic to Indian jujube fruits, whereas the control fruits remained asymptomatic (**Figure 7A**). Three days after inoculation, the inoculated fruits of each fungal strain developed light brown to dark brown lesions at the inoculation area. Five days after inoculation, the diameter of the lesions caused by each tested fungal strain was measured and presented in **Table 3**. The diameter of the lesions for each fungal strain from repeated experiments with ten replicates was found to be normally distributed as confirmed by the Shapiro-Wilk test (*p*=0.01). Thus, to identify statistically significant differences, we conducted ANOVA with subsequent DMRT at a significance level of *p*<0.05. The lesion diameters differed depending on fungal strain. When comparing lesion diameters among the fungal species, *C. siamense* produced the largest lesions, followed by *F. weifangense* and *C. fructicola*, with statistically significant differences observed between species. The largest lesion was observed in jujube fruits inoculated with *C. siamense* strain SDBR-CMU528. Based on these results, *C. siamense* has higher virulence than *F. weifangense* and *C. fructicola*. The anthracnose lesions became soft and water-soaked, and orange conidial masses were observed on the surface of the lesions (**Figures 7B–I**). Complete fruit decay resulting from infection by the anthracnose pathogens was observed, with *C. siamense* causing total fruit rot by seven days after inoculation, whereas *C. fructicola* led to full decay between 12 and 15 days after inoculation. In the case of fruit rot symptoms caused by *F. weifangense*, the lesions became soft and water-soaked as illustrated in **Figure 7J**. These lesions were extensively covered with dense white mycelium. The disease advanced, leading to complete fruit decay between 10 and 14 days after inoculation. Both anthracnose and fruit rot symptoms observed in the experiment were consistent with those typically seen during postharvest storage. The fungi were isolated from each of the inoculated fruits and identified through morphological observation and molecular data as *C*. *fructicola*, *C*. *siamense*, and *F. weifangense*, fulfilling Koch’s postulates.

**Table 3:** The diameter of the lesions caused by each tested fungal strain after five days after inoculation.

Treatment		Diameter of lesions (mm)*
Control		0.00 ± 0.00 h
Inoculation of *Colletotrichum fructicola*	SDBR-CMU527	13.05 ± 1.64 e
	SDBR-CMU531	11.20 ± 1.20 f
	SDBR-CMU532	9.95 ± 1.04 g
Inoculation of *Colletotrichum siamense*	SDBR-CMU528	24.25 ± 0.94 a
	SDBR-CMU529	22.95 ± 1.06 b
	SDBR-CMU530	21.35 ± 1.02 c
	SDBR-CMU533	21.10 ± 1.30 c
	SDBR-CMU534	21.65 ± 1.08 c
Inoculation of *Fusarium weifangense*	SDBR-CMU535	14.85 ± 1.09 d

*Results are expressed as mean ± standard deviation from ten replicates with twice. According to Duncan’s Multiple Range Test (*p*<0.05), distinct letters within the same column are regarded as statistically different.

### 3.5 Effect of commercial fungicides against fungal pathogens

**Figure 8:**
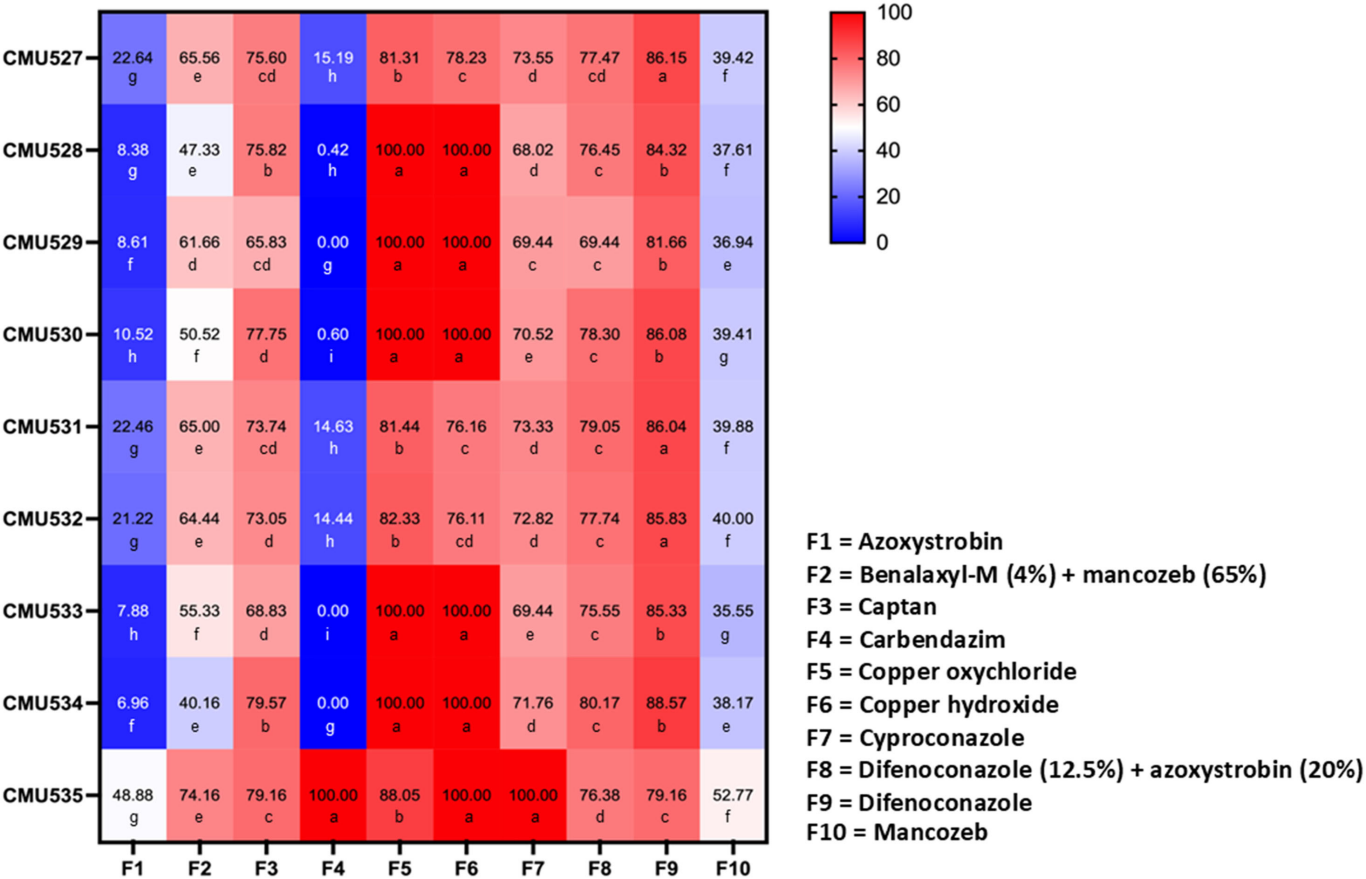
Heatmap showing the percentage of mycelial growth inhibition at the recommended concentration of each fungicide for *C. fructicola* (strains SDBR-CMU527, SDBR-CMU531, and SDBR-CMU532), *C. siamense* (strains SDBR-CMU529, SDBR-CMU530, and SDBR-CMU533), and *F. weifangense* (strain SDBR-CMU535). Data are presented as means from three replicates, each performed twice. Different letters assigned within each fungal strain indicate statistically significant differences (*p*<0.05), as determined by Duncan’s Multiple Range Test.

The response of fungal pathogens to commercial fungicides was assessed based on the percentage of mycelial growth inhibition at the recommended concentration of each fungicide and subsequently classified as completely inhibited (100% inhibition), effective (≥50% inhibition), or ineffective (<50% inhibition). The results indicated that mycelial growth inhibition varied depending on the fungicides, fungal species, and strains, as shown in **Figure 8**. The mycelial inhibition percentages for each fungal strain, when tested against various fungicides, were found to be normally distributed as confirmed by the Shapiro-Wilk test (*p*<0.001). Thus, to identify statistically significant differences, we conducted an ANOVA, with subsequent DMRT at a significance level of *p*<0.05. Among the ten fungicides tested, copper oxychloride and copper hydroxide showed total inhibition (100%) of *C. siamense*. Captan, cyproconazole, difenoconazole + azoxystrobin, and difenoconazole were effective against all tested strains of *C. siamense*. Additionally, benalaxyl-M + mancozeb was effective against *C. siamense* strains SDBR-CMU529, SDBR-CMU530, and SDBR-CMU533. However, none of the fungicides used in this study showed complete (100%) inhibition of *C. fructicola*. It was found that the growth of all strains of *C. fructicola* was suppressed by all fungicides, except azoxystrobin, carbendazim, and mancozeb. Difenoconazole showed the significantly highest growth inhibition for *C. fructicola*. For *F. weifangense*, carbendazim, copper hydroxide, and cyproconazole demonstrated complete (100%) inhibition, while azoxystrobin was ineffective against the growth of this fungus. Copper oxychloride was the fungicide that had the highest growth inhibition value for *F*. *weifangense*, followed by difenoconazole, captan, difenoconazole + azoxystrobin, benalaxyl-M + mancozeb, and mancozeb.

## 4 Discussion

*Colletotrichum* and *Fusarium* are among the most economically important fungal genera globally. They cause significant diseases in many valuable fruit crops, including jujube, that are grown around the world. Specifically, *Colletotrichum* causes anthracnose disease while *Fusarium* is responsible for various rot diseases in different crops (Srivastava and Mehra, 2004; Wang et al., 2005, 2009; Rahman et al., 2011; Zakaria, 2021; Won et al., 2023; Xu et al., 2023). In this study, three fungal strains of *C. fructicola* and five strains of *C. siamense* were obtained from anthracnose lesions, and one strain of *F. weifangense* was isolated from rot lesions on Indian jujube fruits in northern Thailand. The methods used to identify these fungal taxa were similar to those used for *Colletotrichum* and *Fusarium*, combining morphological characterization with multi-gene phylogenetic analysis (Prihastuti et al., 2009; Crous et al., 2021; Mu et al., 2021; Norphanphoun and Hyde, 2023; Suwannarach et al., 2024). Koch’s postulates were confirmed by performing pathogenicity tests on all isolated strains of *C*. *fructicola*, *C*. *siamense*, and *F*. *weifangense*. These findings demonstrate that postharvest anthracnose and rot diseases in Indian jujube fruits from northern Thailand are similar to those caused by previously identified fungal pathogens. Anthracnose caused by *C. gloeosporioides* is a major disease of jujube fruits, significantly reducing both quality and yield worldwide, including in Thailand (Srivastava and Mehra, 2004; Azam-Ali et al., 2006; Athipunyakom et al., 2010; Rahman et al., 2011; Mirzaee, 2014). *Colletotrichum gloeosporioides*, causing anthracnose disease on the fruits of Indian jujube, has been reported in Bangladesh, Brazil, and India (Srivastava and Mehra, 2004; Rahman et al., 2011). In addition, four *Colletotrichum* species, including *C*. *fragariae*, *C. gloeosporioides*, *C. musae* and *C. nymphaeae* have been identified as pathogens of anthracnose fruit rot disease in Chinese jujube (*Z. jujuba*) in South Korea (Kwon et al., 2019; Kang et al., 2023; Won et al., 2023). *Colletotrichum fructicola* and *C. siamense*, which cause anthracnose in Indian jujube fruits, were identified in Taiwan (Duan and Chen, 2022, 2024). In China, *C. siamense* has been identified as a pathogen responsible for anthracnose disease affecting the fruits of both Chinese jujube (Han et al., 2023a) and Indian jujube (Shu et al., 2021). Therefore, this study is the first documented occurrence of *C. fructicola* and *C. siamense* as causal agents of anthracnose disease in Indian jujube fruits in Thailand.

Fruit rot diseases caused by fungi are among the most important diseases affecting jujube fruits. *Alternaria alternata*, *A. tenuissima*, *Botryosphaeria dothidea*, *Cercospora nicotianae*, *Cladosporium* sp., *Diaporthe eres*, *F. sulphureum*, *Nigrospora oryzae*, *Nothophoma quercina*, *Penicillium citrinum*, and *P. corylophilum* have been previously identified as the causative pathogens of fruit rot disease in jujube during postharvest storage conditions (Wang et al., 2005, 2009; Xia et al., 2005; Alam et al., 2017; Xu et al., 2023). In China, four *Fusarium* species, *F. graminearum*, *F. lateritium*, *F. oxysporum*, and *F*. *proliferatum* have been reported as a pathogen causing fruit rot disease in Chinese jujube (Zhang et al., 2012, 2013; Xu et al., 2023). In this study, *F. weifangense* was identified as the causal agent of fruit rot in Indian jujube fruits in Thailand. Prior to this study, *F. weifangense* had been reported as a causal agent of wheat scab disease (Han et al., 2023b) and panicle rot in quinoa (*Chenopodium quinoa*) (Yin et al., 2021). It has also been identified as a plant-associated fungus in several hosts, including alfalfa (*Medicago sativa*) (Xia et al., 2019), Chinese plum (*Prunus salicina*) (Ai et al., 2025), lettuce (*Lactuca sativa*) (Xia et al., 2019), chili (*Capsicum* spp.) (Wang et al., 2019), and chestnut rose (*Rosa roxburghii*) (Zhang et al., 2023). Consequently, this study constitutes the first evidence of *F. weifangense* associated with fruit rot in Indian jujube in Thailand and around the world.

Numerous previous studies have demonstrated that fungicides can effectively inhibit mycelial growth of plant pathogenic fungi, particularly species within the genera *Colletotrichum* and *Fusarium*. These studies have documented varying degrees of inhibition, effective, and ineffective when tested under laboratory conditions (Baria and Rakholiya, 2020; Usman et al., 2021; Li et al., 2023; Karim et al., 2024; Pandey et al., 2024; Suwannarach et al., 2024). In this study, variations in fungal pathogen response to fungicides were observed among different fungicides, species, and individual strains. These findings are consistent with previous studies that showed the type and concentration of each fungicide, as well as the fungal species and strains, influence the mycelial growth response of *Colletotrichum* and *Fusarium* to fungicides (Usman et al., 2021; Karim et al., 2024; Apithanasakulngeon et al., 2025). In the fungicide screening test conducted in this study, copper oxychloride and copper hydroxide achieved complete inhibition of *C. siamense*. Although no fungicide completely inhibited *C. fructicola*, difenoconazole was the most effective, followed by copper oxychloride. These findings are consistent with the use of copper oxychloride in managing anthracnose in jujube (Azam-Ali et al., 2006). All strains of *C. fructicola* and *C. siamense* associated with jujube anthracnose were ineffective to azoxystrobin, carbendazim, and mancozeb. These findings are consistent with previous reports of azoxystrobin ineffectiveness against *C. siamense* strains causing mango anthracnose in Thailand (Kongtragoul et al., 2020). Similarity, carbendazim was highly ineffective against *C. fructicola*, the causal agent of mulberry anthracnose in China (Li et al., 2024). Carbendazim, mancozeb, and prochloraz were effective against *C. fructicola*, the pathogen responsible for leaf spot disease in peach palm (*Bactris gasipaes*) in Thailand (Wonglom et al., 2025). However, azoxystrobin was effective against *C. siamense* strains isolated from orange anthracnose (Kongtragoul et al., 2020). Ishii et al. (2022) found that azoxystrobin, in combination with n-propyl gallate, completely controlled *C. fructicola* and *C. siamense*. Apithanasakulngeon et al. (2025) also found that difenoconazole, mancozeb, and prochloraz were effective against *C. siamense* strains causing durian anthracnose in Thailand, but chlorothalonil and pyraclostrobin were ineffective against their growth. Additionally, prochloraz was highly effective against *C. fructicola* and *C. siamense*, which cause peach and walnut anthracnoses in China (Usman et al., 2021; Li et al., 2023). In this study, carbendazim, copper hydroxide, and cyproconazole completely inhibited *F*. *weifangense*, a member of the *F. incarnatum-equiseti* species complex, which also showed high inhibition by copper oxychloride. Similarly, copper oxychloride showed strong effectiveness against mycelial growth of members of the *F. incarnatum-equiseti* species complex, including *F. compactum*, *F. mianyangense*, *F. jinanense*, and *F. sulawesiense* (Suwannarach et al., 2024). Carbendazim showed effectiveness against *F. incarnatum*, which causes papaya fruit rot in India (Bachkar et al., 2021). Furthermore, carbendazim, copper oxychloride, and mancozeb showed high effectiveness against *F. musae*, which causes banana fruit rot (Baria and Rakholiya, 2020). Nevertheless, copper oxychloride showed ineffectiveness against most strains of *F. concentricum*, *F. fujikuroi*, *F. oxysporum*, and *F. solani*, which are responsible for tea dieback disease in India (Pandey et al., 2024). Because fungicides with specific modes of action are highly effective in controlling fungal diseases, they are frequently used by farmers; therefore, accurate identification of pathogens and understanding their response to these fungicides are essential for preventing crop loss (Corkley et al., 2021; Yin et al., 2023; FRAC, 2024). Although this study assessed fungicide response based on mycelial growth inhibition of *C. fructicola* and *C. siamense*, responsible for postharvest anthracnose, and *F. weifangense*, which causes fruit rot in Indian jujube at recommended concentrations, more precise and comprehensive evaluations, such as determining the half-maximal effective concentration (EC_50_), evaluating a broader range of fungicide classes, and conducting conidial germination assays, should be included in future studies to gain deeper insights into fungicidal efficacy. These approaches would ultimately contribute to more effective and sustainable disease management strategies for Indian jujube both in Thailand and globally.

## 5 Conclusions

Postharvest diseases of Indian jujube, caused by *Colletotrichum* and *Fusarium*, are widespread problems that frequently occur during storage. In the present study, *C. fructicola* and *C. siamense* were isolated from anthracnose lesions, and *F. weifangense* was isolated from fruit rot lesions on Indian jujube fruits in northern Thailand. The fungal pathogens were identified through a combination of morphological examination and multi-gene phylogenetic analyses. Pathogenicity tests were conducted, and the results demonstrated that the isolated fungi were capable of causing anthracnose and fruit rot symptoms in inoculated Indian jujube fruits, which resembled those typically observed during postharvest storage. Consequently, this study is the first to identify *F. weifangense* as a novel causative agent of postharvest fruit rot diseases in Indian jujube in Thailand and worldwide. This study also represents the first case of postharvest anthracnose on Indian jujube fruits caused by *C. fructicola* and *C. siamense* in Thailand. The fungicide response assay revealed that both copper oxychloride and copper hydroxide completely inhibited the growth of *C. siamense*. Although no fungicide completely inhibited *C. fructicola*, difenoconazole was the most effective against this species. Carbendazim, copper hydroxide, and cyproconazole all completely inhibited *F. weifangense*. Consequently, the findings of this study enhance our understanding of postharvest anthracnose and fruit rot diseases in Indian jujube, offering critical insights for developing more effective disease management and prevention approaches. The epidemiology of postharvest diseases in Indian jujube fruit across various regions of Thailand, along with the identification of infection sources and the development of effective disease control strategies, will be the focus of our future research.

## Data Availability

The original contributions presented in the study are included in the article/supplementary material. Further inquiries can be directed to the corresponding author.
